# A statistical and biological response to an informatics appraisal of healthy aging gene signatures

**DOI:** 10.1186/s13059-019-1734-z

**Published:** 2019-08-02

**Authors:** James A. Timmons, Iain J. Gallagher, Sanjana Sood, Bethan Phillips, Hannah Crossland, Robert Howard, William E. Kraus, Philip J. Atherton

**Affiliations:** 1 Kings College London, London, UK; 2 University of Nottingham, Nottingham, UK; 3 University College London, London, UK; 4 Duke University, Durham, USA; 5 University of Stirling, Stirling, UK

## Abstract

Jacob and Speed did not identify even a single example of a ‘150-gene-set’ that was statistically significant at classifying Alzheimer’s disease (AD) samples, or age in independent studies. We attempt to clarify the various misunderstandings, below.

**Electronic supplementary material:**

The online version of this article (10.1186/s13059-019-1734-z) contains supplementary material, which is available to authorized users.

In 2013 we discovered a 150 RNA ‘gene-set’ from healthy skeletal muscle (others named HAG) that proved to be a high performing, statistically significant, *non-linear* age classifier of multiple types of human tissue [1]. We profiled muscle samples from an independent birth cohort and found our HAG score correlated with ‘successful aging’ over two decades, using rank-order methods which incorporated the *direction* of gene expression change from the original model [1]. The following year, we found that the HAG score was regulated with age in human hippocampus and in human blood profiles from AD [1]. Since then over 50 HAG have been linked, by other laboratories, to the biology of age and dementia (see Additional file 1), robust confirmation of the biological validity of the HAG. We illustrated the potential utility of the HAG, by combining it with a set of AD regulated ‘disease’ genes [2] to yield a test that distinguished AD samples from *age*- and *gender*-matched controls (Sood et al Fig. 5 [1]). The original disease signature (the Lunnon et al AD signature [2]) was not statistically significant, on its own, in an independent AD cohort. Notably this 150 probe-set plus 48 gene prototype ‘AD blood assay’ (red-dot, Age+AD-disease) out performs all 150 gene-sets presented by Jacob and Speed (Figure 1a) and remains one of the only RNA assays validated in an independent AD cohort. We presented our transcriptomic age model as a logical starting point for machine learning requiring independent data, to produce a tool to facilitate clinical AD research (i.e. screen for accelerated ‘aging’, *the* risk factor for AD).

**Fig. 1:**
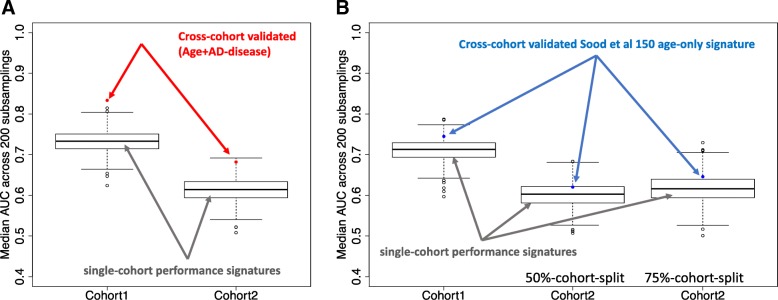
A guide to the graphing layouts produced by Jacob and Speed and bench-marking against our top-ranked AD signature. **a** Using the same code and sampling procedure as Jacob and Speed, we compare the top-ranked gene-set from Fig. 5 Sood et al. [1] with their performance of their random process. Critically, Jacob’s code creates two random sampling objects per R session – rand.sig.1 for GSE63060 and rand.sig.2 for GSE63061, reflecting the different gene content of the GSE63060 and GSE63061 arrays. Jacob and Speed do not implement any cross-cohort validation nor any consideration of correction for multiple testing. Notably, our top-ranked blood signature exceeds random sampling by a substantial margin and critically is ‘active’ in both blood data-sets. Note that their ‘random’ gene-lists are selected from entire gene-chip which contains thousands of published age and AD correlated genes (as listed in our 2015 article, supplementary information). **b** Using the same code and sampling procedure as Jacob and Speed, we present the performance of just the 150 tissue age genes from Sood et al. (blue dot) in blood. We then assess one example of choices made by Jacob and Speed, namely using 50 vs 75% cohort split during withincohort cross-validation. By selecting a 50% split the relative (to ‘random’) performance of our age-150 gene list is impaired. Since the Sood age-150 was never stated to be the only age classifier, we made a genuine attempt at sampling at random by removing known Age (and AD) genes from the sampling pool (as listed in our 2015 article, supplementary information [1]). As Jacob’s code does not implement any cross-cohort validation nor any consideration of correction for multiple testing, our age signature remains the only signature validated in two (blood) cohorts (and across other independent data)

Substantial misunderstandings of our article, by Jacob and Speed, led to them create a narrative through reference to Ein-Dor et al [Ref 2 in their letter], that our work was an example of a classification solution found using a small clinical cohort [3]. Typically such solutions do not transfer to independent clinical cohorts, because the model is over-fitted to *all* the characteristics of the first data set. Notably it was impossible for the HAG signature to belong to this category, as we did not select our gene list using an AD cohort. Importantly, our single HAG ‘150 gene list’ was confirmed to be statistically significant in two independent AD cohorts [1]. In contrast, Jacob and Speed calculated thousands of ROC values; did not apply any statistical test for significance, and never confirmed the performance of any of their gene lists in any independent cohorts. For clarity on this complex topic, Table 1 contrasts some of the major differences in approach and rigor, taken by our team versus the approach presented by Jacob and Speed. It is noteworthy that the approach we took is far more laborious, and we understand why Jacob and Speed may have chosen to skip over most of the following time consuming and important steps.

**Table 1:** A comparison of the approaches of Sood et al. and Jacob and Speed

Performance criteria	Sood et al.	Jacob and Speed
Hypothesis driven gene selection in independent samples	Yes	No
Tissue age ROC performance externally validated	Yes	No
Multi-tissue age classification achieved	Yes	No
Longitudinal health association achieved	Yes	No
Top age gene-set statistically tested in blood	Yes	No
ROC performance assessed within an AD cohort	Yes	Yes
Top gene-set statistically significant across AD cohorts	Yes	No
Top gene-set independently linked to age and dementia	Yes	No
Gene-set trained in AD clinical cohort	No	Yes

To revisit Jacob and Speed analysis we used their computational code, and found that their *within-cohort* modeling of the AD data (an approach we did not use as it is unreliable [4] (Table 1)) yielded 150 gene-sets with performance that reflected laboratory batch-noise within the AD Illumina blood RNA arrays (AdNeuroMed consortium [2]). We illustrate this, by sampling using their code, but only from non-expressed (‘background’) genes (Additional file 1: Figure S1). This noise is unfortunate, but the illustration demonstrates that the performance of their random sampling protocol was not driven, as claimed, to ‘interconnected biology’ or ‘shared gene expression variance’. Furthermore, their claim that our 150-gene age signature (Fig. 1b, blue-dot) lay ‘within’ the performance range obtained via random sampling in blood is neither correct nor a fair like-for-like statistical comparison [4]. Our single 150 HAG test was statistically significant in two independent AD cohorts, and exceeded any background noise. Jacob and Speed random gene-sets require statistical correction for thousands of multiple-tests and notably they did not present any statistical significance values. The importance of our approach, using hypothesis driven signatures and robust external validation (i.e. using independent data to validate a model) over their ‘easy to perform’ within data sampling is neither controversial or recent knowledge - See Konig et al. and cited articles [4].

We also found that their (less reliable) ‘within cohort’ ROC performance approach was enhanced by some of their choices e.g. 50% data-splits resulted in ~ 10% gain in ROC value, in their favor (Fig. 1b). Their random gene lists were not actually ‘random’ from a biological perspective. Sampling occurred from known age and AD correlated genes (see methods). Removal of age and AD genes known at the time of our study (2015) also reduces the performance of sampling at random (Additional file 1: Figure S2). Curiously, Jacob and Speed did not match AD cases with controls, for chronological age and gender (the two greatest risk factors for AD). Instead they combined AD with the Mild Cognitive Impairment (MCI) samples - a heterogenous population, comprising several clinical conditions and many who never develop AD [5]. This was clinically invalid but also unnecessary, as all required information was included in the GEO repository e.g. age, gender and clinical sample status (embedded in GSE63060 and GSE63061 as visualized in his files supplied with their letter (Additional file 1: Figure S3)). Combining the AD and MCI clinical samples also exaggerated the *within* cohort performance of their approach while it impaired the performance of our top-ranked gene-set (Additional file 1: Figure S4). Critically Jacob and Speed should have been able to replicate the design of our study analysis as all data necessary was at GEO, as illustrated in Additional file 1: Figure S3 - a screen shot taken directly from implementing the code provided by Jacob and Speed.

Finally, Jacob and Speed have claimed that our tissue age model was itself ‘unremarkable’. A clue to the exceptional performance of our age signature was the observation that when using muscle RNA profiles as the external validation data (classification space), the age of brain samples was classified correctly [1]. However, we formally revisited this issue by comparing our [1] HAG signature with 10,000 gene-sets chosen at random from the same muscle cohort. We calculated performance of each recorded ‘at random’ gene-set, using *cross-cohort* ‘gold standard’ external validation (four *independent* muscle data-sets; code and methods are included in the Additional file 1). Our 150 HAG signature was ranked better than all 10,000 ‘random’ gene-sets (Additional file 1: Figure S5A). We calculated the statistical significance of the average ‘random’ gene-set, and the performance of an ‘age model selected at random’ was not significant (*unadjusted p* = 0.113), despite having an ROC value > 0.6. Even gene-sets discovered and tested *within* a single cohort were largely inferior to our age signature (Additional file 1: Figure S5B). Our observations lead to the conclusion that the claims made by Jacob and Speed are flawed, at least in part because they did not present a single example of a new 150-gene-set that significantly worked *across* independent data as a tissue age classifer, a muscle-based health prognostic or a blood AD classifier. Thus, while we agree with Ein-Dor et al, that classifiers built on small clinical cohorts should be treated with caution; reflecting the interactive network of tissue and cellular gene expression [6] and simple technical factors such as noise, as we did not build a disease classifier using AD or any other disease samples, we find Jacob and Speed's criticism [3] of our work unfounded.

## Additional file

Additional file 1: Supplementary figures S1-S5, Supplementary Methods and code. (DOCX 3780 kb)
