# Supplementary material for: A statistical and biological response to an informatics appraisal of healthy aging gene signatures
Source: Genome Biol. 2019 Aug 2;20:152. doi: 10.1186/s13059-019-1734-z (PMC6676519; doi:10.1186/s13059-019-1734-z)
Supplement: Supplementary file 1 — Supplementary figures S1-S5, Supplementary Methods and code. (DOCX 3780 kb) [file 13059_2019_1734_MOESM1_ESM.docx]

**Supplementary Results and Methods**

**Cross-cohort validated (Age+AD-disease)**

**Fig S1. Demonstrating that Jacob and Speed were studying noise.** Using the code and procedure provided by Jacob and Speed, we sampled at random from the 10,000 probes on the array that corresponded with genes expressed at background levels i.e. not significantly expressed in blood according to the Illumina microarray technology (mean intensity <80 units and p=0.5 for detection call). Their sampling procedure, applied to genuine AD versus control samples, demonstrates that within cohort random performance reflects nothing more than laboratory noise. Note that these noise-driven random gene-sets represent only *within* cohort validation (Jacob’s code creates two random sampling objects in the R session – rand.sig.1 for GSE63060 and rand.sig.2 for GSE63061, reflecting the different gene content on GSE63060 and GSE63061 arrays). Each is a unique list, there is no cross-cohort validation and no test of statistical significance. It is not appropriate to present of a cross-cohort validated and statistically significant hypothesis driven single 150-gene-set signature, with their ‘within cohort’ ROC performance descriptive metrics as generated by their sampling process. In fact, the marked difference between average AUC in cohort 1 and cohort 2, during random sampling, should have alerted Jacob and Speed to this issue. As before, our top-ranked across-cohort validated signature (red-dot) remains far above the performance achieved through sampling at random.

**Fig S2. Exploring a biological approach to sampling at random.** Using the code and procedure provided by Jacob and Speed, we all known age and ad genes as of the time of our publication (2015) to simulate, from a biological perspective, a genuine attempt to bench-mark the performance of ‘random gene sets’. Note, because a major driver to the random performance in blood is batch noise within the microarrays provided by Lovestone, Hodges and Lunnon (Fig S1) this sort of analysis over estimates the performance possible through ‘sampling at random’ from genes unelated to age or ad (as does relying on only within-cohort validation). Thus, even with a background of substantial batch-noise, removing known AD and age genes reduced within cohort ‘random’ sampling performance by further ~0.02 AUC units i.e. ~7.5%. As before, our top-ranked across-cohort validated signature (red-dot) remains far above the performance achieved through sampling at random. Note, the performance of our gene-set (red-dot) remains unoptimized and thus the starting point for further development as part of a broader strategy (as clearly and extensively discussed in Sood et al [1] from pages 10 to 12).

**Fig S3. A screen shot from the R-Studio session relying on the files provided by Jacob**. In the right-hand side panel, a list of objects in memory for the particular R session. Within the object GSE63060.annot (annotation information for cohort 1) and GSE63061.annot (annotation information for cohort 2) all of the information required to create an age and gender matched grouping of AD versus Control can be found (red-ovals). This information comes, in turn, directly from the GEO file we deposited at the NIH website in 2015 and Jacob used for all of his ‘analysis’. There was therefore absolutely no need to combine AD and MCI sub-groups. Further, as Jacob and Speed create these two objects to then identify AD, MCI and Control samples (and combine AD with MCI) and this requires direct inspection of the list structure to identify which information to select on. All of the appropriate information, to specifically study AD samples, were available on GEO.

**Fig S4. Use of the wrong clinical samples, and without age or gender matching samples.** Using exactly the same code and sampling procedure as Jacob and Speed (i.e. sampling from all genes including known AD & age genes, and ignoring the lack of cross-cohort validation for now), we examine the impact of their choice of combining AD and MCI samples, versus analysis that compares genuine AD versus control samples (balanced for age and gender). Critically Jacob and Speed should have been able to replicate the design of our study analysis as all data necessary was at GEO, as illustrated in Fig S3 - a screen shot taken directly from implementing the code provided by Jacob and Speed. The only approach that is valid (age and gender matched AD samples) reduces the performance of ‘random’ sampling by ~0.025 AUC units and improves the performance of our gene-set i.e. their invalid grouping produced 10% net relative improvement in performance across the two cohorts. The performance of their approach was further exaggerated by failing to calculate an average AUC by external validation, or other variables such as graphing choices.

**Figure S5. Naïve within-cohort classification performance is unreliable for predicting which gene-set will on average validate in multiple independent data-sets.** Jacob and Speed claim that our 150-gene age classifier is not distinct. Their conclusion reflects ignoring the first rule of classification analysis i.e. the same gene-set should give good performance across multiple data-sets. To illustrate the difference between within-cohort and across cohort performance we sampled at random (10,000 times) from the genes on the U133+2 chip. We checked the performance of each individual signature in 4 studies using external validation (i.e. a fifth independent cohort represented the known (young or old) samples). This type of gold-standard check, is something that even within-cohort and leave-out cross validation methods can never address as it can’t distinguish common biology from shared (non-transferable) technical variance. Naïve within-cohort analysis – plotting the Sood et al age signature (blue dot) versus random sampling within each cohort (none of which were used to selected the Sood 150 signature – that was selected via a hypothesis driven model of ‘healthy tissue ageing’) – observes a few examples of random gene-sets that appear to match our 150 gene-set. If the average performance across all independent data is considered this is no longer the case – none of the ‘sporadic’ or ‘study specific’ gene-sets work in other data sufficiently well to be meaningful. This is illustrated in the ‘gold-standard across cohort’ plot where the Sood et al [1] 150-age gene-set is ranked first out of all 10,000 gene-sets sampled at random and the average random performance is < 0.7 AUC and is not statistically significant across studies (*unadjusted* p=0.113).

**Sood et al 150 age signature and direct links to longevity and dementia**

Since demonstrating that we had a robust binary RNA diagnostic (150 genes) of human tissue we have demonstrated [2] that a small part of the gene signature (genes selected by PCA in muscle) is regulated by mTOR activity *in vitro,* linking the signature to a central regulator of longevity and metabolism across many lower species [3]. Remarkably, there are now more than >50 genes from our 2013 prototype now linked to aging and dementia by independent laboratories - more than any other human age-signature we are aware of [4,5,14–23,6,24–33,7,34–43,8,44–53,9,54–63,10,64,11–13]. A few additional genes are directly involved with neurogenesis [48,65,66] or other pathways indirectly linked to aging or neurobiology (e.g. mTORC-ubiquitin). A further 9 were already linked to ageing or dementia at the time we published [67,68,77–83,69–76]. This independent work demonstrates that the biology of the 150 age signature is anything but random – and in fact links directly to longevity mechanisms and dementia.

**Methods**

**Replication of Jacob and Speed’s analysis**

We utilized the code and data provided by Jacob online, to evaluate the different options they chose to make, when carrying out their analysis. Each choice of setting in their code required analysis to run for weeks at a time and it took >3 months to carry out each of the comparison required to consider the options that Jacob and Speed had or had not considered during the 3yr period to looked at our study. When removing the known (published at the time of our article) age and AD related genes, we simply had to filter one of the objects created by Jacob and Speed’s code. The same line was utilized to remove/or include (using positive selection of the remaining genes) target genes for the Jacob and Speed’s code then to sample from. For example, using a text file with a single list of genes (filter) we could then retain only the Illumina probes expressed with a background level signal (noise). In other cases, we removed just a small subset (e.g. known age and AD genes), leaving the majority of the Illumina probes for sampling. The additional line of code required to achieve this simple filter step was:

**1) Read in a list of genes (in this case probe-sets) and run 1 extra line of code**

**X.GSE59880.filtered** <- X.GSE59880[,-which(colnames(X.GSE59880) %in%rcsv$HU133.Probeset_ID)]

**2) Use the X.GSE59880.filtered object in line 102 of the Jacob and Speed code in their R script 'ad-subsample.R', instead of using X.GSE59880 (which represents all genes on the chip, including our age genes)**

rand.sig.affy <- sample(colnames(**X.GSE59880.filtered**), 150)

It is trivial to locate the point in the code to use this step by searching the R code for the object being filtered e.g. ‘X.GSE59880’. We also provide a working version of Jacob and Speed’s code to further highlight where the additional minor step is used – as well as some commentary on the unorthodox coding produced by Jacob and Speed (e.g. where they re-cycle R-object names making their code potentially unreliable to an inexperienced user). All examination and subsequent analysis using the code provided by Jacob and Speed was carried out by James Timmons and by Iain Gallagher and other colleagues - while the results were critiqued by all of the authors, with specific focus on components that reflected their own expertise (e.g. biology, clinical, statistical) and role in the project.

Thus, use of Jacob and Speed’s code took ~ 3months and increased the electricity usage in the office by ~300% (reflecting the energy intensive nature of 24hr random sampling protocols). Energy plot available on request. The analysis was run on a 64GB RAM 2013 8-core Mac-Pro (no parallel processing code). All gene lists and other large files (R sessions, >1GB) are available on request.

All code can be found below and all other required details or code are presented by Jacob and Speed.

**Our genuine and robust random sampling versus Sood 150 for muscle age analysis methods**

We also carried our random sampling however our method not only created random gene-sets but recorded the individual performance and identity of each ‘random’ gene-set so it could be used across independent data-sets. We provide code and describe a simple process for assessing the results of random sampling, across multiple independent data-sets using gold-standard external validation. First, In order to demonstrate the performance of random gene-sets as muscle tissue age classifiers we first removed the genes we had originally identified with classification ability in ageing (n=670) [1] from the starting pool of genes to be sampled. We then evaluated the ability to classify, with statistical significance age in 10,000 ‘random gene-sets’ of 150 genes. Importantly, unlike Jacob and Speed we evaluated the same set of 10,000 different 150 gene-set lists across all data examined. Classification performance was assessed using accepted methods, as reflected on by Speed himself at an earlier time [84], with external validation so that each gene-set is judged in an independent data set (see below). This was contrasted with LOOCV carried out and plotted for naïve within cohort performance (Figure S4). This work was carried out by Sanjana Sood and code checked by Iain Gallagher.

**A random sampling code followed by data plotting from the saved sessions**

#----------------------------------------------------------------------------#

#KNN Validation using Independent external validations

#----------------------------------------------------------------------------#

rm(list=ls())

library("inSilicoDb")

library("inSilicoMerging")

library(affy)

library(class)

library(limma)

library(frma)

library(ROCR)

setwd('~/Desktop/Sanjana/Projects/Independent validation/')

name<-"Derby"

#get the data

dataCampbell <- ReadAffy(celfile.path='~/Desktop/Sanjana/Projects/Independent validation/CEL files/Campbell/')

datatest <- ReadAffy(celfile.path='~/Desktop/Sanjana/Projects/Independent validation/CEL files/Derby/')

######## Makes all Data Sets Annotations Consistent ########

sampleNames(dataCampbell) <- sub('\\.CEL$', '', sampleNames(dataCampbell))# .CEL off names

sampleNames(dataCampbell) <- sub('\\.cel', '', sampleNames(dataCampbell))# .CEL off names $doesnt make any difference

phenoTabletrain <- read.table('Phenodata/phenoData_Campbell.csv', sep=',', header=T)

rownames(phenoTabletrain)<-phenoTabletrain$array

mt_train<-match(sampleNames(dataCampbell), rownames(phenoTabletrain))

phenoData(dataCampbell) = new('AnnotatedDataFrame', data = phenoTabletrain[mt_train,])

sampleNames(datatest) <- sub('\\.CEL', '', sampleNames(datatest))# .CEL off names

sampleNames(datatest) <- sub('\\.cel', '', sampleNames(datatest))# .CEL off names $doesnt make any difference

phenoTabletest<- read.table('Phenodata/phenodata_Derby.csv', sep=',', header=T)

rownames(phenoTabletest)<-phenoTabletest$array

mt_test<-match(sampleNames(datatest), rownames(phenoTabletest))

phenoData(datatest) = new('AnnotatedDataFrame', data = phenoTabletest[mt_test,])

#-------------------------------------------------------------------------------

# Create Merged Data Set with preprocessing using frma and without batch effect removal

#-------------------------------------------------------------------------------

esettrain<- frma(dataCampbell)

esettest<- frma(datatest) # frma = Preprocessing and analysis for single microarrays and microarray batches

esets <- list(esettrain, esettest)

#--------------------------------------------------------------------------------------

# Create Merged Data Set with preprocessing and with batch effect removal using E Bayes

#---------------------------------------------------------------------------------------

esetMerge1 <- merge(esets, method = "COMBAT") # Merge them using 'COMBAT also called EBayes

expMerged <- exprs(esetMerge1)

# number of neighbours to consider

knn.k <- 3

mt_train1 <- which(esetMerge1$dataset=="Campbell")

mt_test1 <- which(esetMerge1$dataset=="Derby")

expr.train <- exprs(esetMerge1[,mt_train1]) ## expression values of a particular data set to proceed as training set

expr.test<- exprs(esetMerge1[,mt_test1])## expression values of a particular data set to proceed as test set

training.groups <- factor(as.numeric(pData(esetMerge1[,mt_train1])$group)) # group names of training samples

testing.groups <-factor(as.numeric(pData(esetMerge1[,mt_test1])$group)) # group names of test samples

# Create objects to save validation performance

n_runs<-10000 # number of Random sampling runs

sens <- rep(0,n_runs)

spec <- rep(0,n_runs)

percentage<-rep(0,n_runs)

auc<-rep(0,n_runs)

##### run line 78 to 92 ONLY ONCE and for remaining EV runs for other muscle datasets comment this section out as we want to use same 10000 RS lists across all muscle datasets

## create an object with all the probesets on the microarray platform

scoreMatrix<-rownames(expMerged)

## read the file with all the known age genes/probesets

age_ps<-read.csv("~/Desktop/Sanjana/Projects/Independent validation/age_genes_all.csv",sep=",",header = T)

remove<-match(age_ps$HGU133Plus2,scoreMatrix)

##remove them from the list from which random sampling will be done

scoreMatrix_without_age<-scoreMatrix[-remove]

## create a matrix of randomly sampled 150 ps to be used across EV of datasets

probe_list<-matrix(nrow=n_runs,ncol=150)

for (j in 1:n_runs){

rnd_ids <- as.character(sample(scoreMatrix_without_age, 150))

probe_list[j,]<-rnd_ids

}

# Carry out EV with 10000 random sample of 150 ps and record there performance

for (i in 1:n_runs){

train.genes <- expr.train[rownames(expr.train) %in% probe_list[i,], ] # get probe sets measures from training samples

test.genes <- expr.test[rownames(expr.test) %in% probe_list[i,], ] # get 150 probe set measures from expr.test (test samples)

training.data <- t(train.genes) # transpose for knn

#will store the final results for each array

validation.score <- matrix(ncol=2,nrow=ncol(expr.test))

# knn validation using new dataset and common genes

# Out of n new arrays use n-1 arrays for training and 1 for testing and record success/failure for each test

#------------------------------------------------------------------------------------------------------------------

# Roc Analysis: Assign '-1' to OLD sample (i.e.Negative Case) and '1' to Young (Positive Case) of Test Data Set

#-----------------------------------------------------------------------------------------------------------------

rocScore <- matrix(ncol=2,nrow=ncol(expr.test)) # to process ROC analysis

colnames(rocScore) <- c("label","predict")

labels <-testing.groups

##### Assigns'-1' for OLD sample and '1' for Young in column label of rocScore

for(abc in 1:ncol(expr.test)){

if(labels[abc] == 1)

rocScore[abc,1] <- -1

else if(labels[abc] == 2)

rocScore[abc,1] <- 1

}

record <- 0

for (validation.array in 1:ncol(expr.test)){

test.data <-t(test.genes[,validation.array]) # knn test set case (1 test sample at a time)

predict <- knn(train=training.data, test=test.data,cl=training.groups, k=knn.k,prob=TRUE) ### KNN prediction

if(predict==1){

rocScore[validation.array,2] <- -1} else if(predict==2)

rocScore[validation.array,2] <- 1

if (predict==testing.groups[validation.array]){

validation.score[validation.array,1] <- colnames(expr.test)[validation.array] # predicted sample name

validation.score[validation.array,2] <- "Correct Prediction"

record <- record+1

}

else {

validation.score[validation.array,1] <- colnames(expr.test)[validation.array]

validation.score[validation.array,2] <- "Incorrect Prediction"

}

}

percentage[i] <- 0

percentage[i] <- ceiling((record/validation.array)*100) #calculates their performance (i.e. the percentage of correct predictions)

#------------------------------------------------------------------------------------------------------------

# Calculating the Values of ROC Curve

#-----------------------------------------------------------------------------------------------------------

threshold <- 0

binary.labels <- rocScore[,1] == 1 # assigns TRUE if to be predicted value is 1 (i.e young) & assign FALSE otherwise

#### '1' used for Young sample and '-1' for Old sample

tp <- sum((rocScore[,2] > threshold) & binary.labels) # calculates total number of True Positives, i.e young classified as young

sensitivity <- tp/sum(binary.labels) # calculates sensitivity i.e TP/total Positives from class labels

fp <- sum((rocScore[,2] > threshold ) & (!binary.labels)) # calculates false positives for FPR/'1-specificity'

fpRate <- fp/sum(!binary.labels) # 1-specificity

sens[i]<- round(sensitivity, digits = 3) ## rounding off

FPR <- round(fpRate, digits = 3) ## rounding off

spec[i]<-1-FPR

pred<-prediction(rocScore[,2], rocScore[,1])

auc[i]<-attributes(performance(pred, 'auc'))$y.values[[1]]

cat("Performance of the",i, "Generation = ",percentage[i],"Spec=",spec[i],"Sens=",sens[i],"\n")

}

# Create the final output matrix

output<-as.data.frame(cbind(percentage,spec,sens,auc,probe_list))

colnames(output)<-c("percentage","specificity","sensitivity","auc",1:150)

min(auc)

max(auc)

write.table(output, paste(name,"Randomsampling_External_validation(KNN).csv",sep=""),sep=',',quote=F,row.names = T)

# go back to line 17 and rerun the analysis for other muscle datasets and make sure to comment line 78-92

########## making the final data plot from multiple runs of analysis #################################################################################

**#Read the data matrices with Random sampling output that you have saved on line 183 from all studies you have analysed**

Trappe<-read.table("TrappeRandomsampling_External_validation(KNN).csv",sep=",",header=T)

Hoffmann<-read.table("HoffmanRandomsampling_External_validation(KNN).csv",sep=",",header=T)

Derby<-read.table("DerbyRandomsampling_External_validation(KNN).csv",sep=",",header=T)

Kraus<-read.table("KrausRandomsampling_External_validation(KNN).csv",sep=",",header=T)

t_auc<-Trappe$auc

d_auc<-Derby$auc

h_auc<-Hoffmann$auc

k_auc<-Kraus$auc

random_sample_auc<-as.matrix(cbind(t_auc,h_auc,d_auc,k_auc))

# auc for EV of these signatures has been calculated separately for trappe,hoffman,Derby,Kraus by running the script for the other data sets

trappe_rank<-rank(random_sample_auc [,1])

hoffman_rank<-rank(random_sample_auc [,2])

derby_rank<-rank(random_sample_auc [,3])

kraus_rank<-rank(random_sample_auc [,4])

rank_data<-as.matrix(cbind(trappe_rank,hoffman_rank,derby_rank,kraus_rank))

cumulative<-apply(rank_data,1,median)

final_plot<-as.data.frame(cbind(rank_data,cumulative))

write.table(final_plot,"Final_cumulativerank_allsigs_EV.csv",sep=",",quote=F,row.names = F)

**Our one data-set at a time external with LOOCV code**

---

title: "Independent Validation"

output: pdf_document

---

Having previously identified a set of genes able to classify tissues as having a young or old profile we now examine the ability of this 'geneset' to classify independent datasets of young vs old tissue samples (samples not used in generating the classification model). The original data used to select the genes is not used at ANY stage of this subsequent process

We first clear the workspace, load required libraries and set some pointers to directories containing the data required.

```{r eval=FALSE}

rm(list=ls())

library(inSilicoDb)

library(inSilicoMerging)

library(affy)

library(class)

library(limma)

library(frma)

library(ROCR)

#Set pathway to CEL files

pathC = 'Path/training_set'

pathM = 'Path/test_set'

#Set names

Training_data<-'training_set_name'

Test_data<-'test_set_name'

```

The data to be classified is loaded. These are microarray cel files.

```{r eval=FALSE}

dataC <- ReadAffy(celfile.path=pathC)

dataM <- ReadAffy(celfile.path=pathM)

```

The phenotype data is loaded from an external file. This file contains the array identifiers as well as phenotype information such as group membership. We use the array name to ensure that the rows of the phenotype data match the order of the arrays.

```{r eval=FALSE}

sampleNames(dataM) <- sub('\\.CEL$', '', sampleNames(dataM))

phenoTableM <- read.table('Phenodata/training_set.csv', sep=',', header=T)

rownames(phenoTableM) <- phenoTableM$array

mtM <- match(sampleNames(dataM), rownames(phenoTableM))

sampleNames(dataC) <- sub('\\.CEL$', '', sampleNames(dataC))

phenoTableC <- read.table('Phenodata/test_set.csv', sep=',', header=T)

rownames(phenoTableC)<-phenoTableC$array

mtC<-match(sampleNames(dataC), rownames(phenoTableC))

# attach the pheno data

phenoData(dataM) = new('AnnotatedDataFrame', data = phenoTableM[mtM,])

phenoData(dataC) = new('AnnotatedDataFrame', data = phenoTableC[mtC,])

```

The microarray data we will use to validate our classifier were generated in different laboratories at different times and are independent biologically and from a technical perspective (including gene-chip format). The different sources of data can introduce technical variance that does not reflect the biological experiment. Below we use the [```fRMA```](http://biostatistics.oxfordjournals.org/content/11/2/242.abstract) algorithm to limit the influence of technical variance e.g. different batches of microarrays. The technical manual for ```fRMA``` is [here](http://www.bioconductor.org/packages/release/bioc/html/frma.html).

```{r eval=FALSE}

esetC <- frma(dataC)

esetM <- frma(dataM)

```

The ```fRMA``` datasets are adjusted using the [```COMBAT```](http://biostatistics.oxfordjournals.org/content/8/1/118.abstract) method which also corrects for batch effects across the the separate microarray datasets. After this treatment the adjusted datasets are prepared for the assessment of classification performance.

```{r eval=FALSE}

esets <- list(esetC, esetM)

esetMerge1 <- merge(esets, method = "COMBAT")

mtC <- which(esetMerge1$dataset=="training_set")

mtM <- which(esetMerge1$dataset=="test_set")

```

We use the knn classifier with a constant k=5 to examine the performance of our classifying geneset on independent microarray datasets. The strategy here is to use a NEW microarray dataset as the 'expression space' (named 'train' in the code) for predicting one sample at a time from the new 'test' batch of microarray data.

In this section we also create the data structures required for later receiver operator curve (ROC) analysis of the results. Specifically we set up a two column matrix with columns for the actual class (```label```) of each case and the prediction made by the knn classifier (```predict```). Label '1' is assigned to a case if it is 'young' and '-1' if it is 'old'.

Using the training and testing data we first extract only expression data for the previously selected age classifier geneset (which remains a fixed variable).

```{r eval=FALSE}

knn.k <- 5

#Load previously identified classification genes

scoreMatrix<-read.table("genes.txt",sep='\t', header=T)

rownames(scoreMatrix) <- scoreMatrix[,1]

#train data

expr.train <- exprs(esetMerge1[,mtC])

train.genes <- expr.train[rownames(expr.train) %in% rownames(scoreMatrix), ]

training.groups <- factor(as.numeric(pData(esetMerge1[,mtC])$group))

#test data

expr.test<- exprs(esetMerge1[,mtM])

test.genes <- expr.test[rownames(expr.test) %in% rownames(scoreMatrix), ]

testing.groups <-factor(as.numeric(pData(esetMerge1[,mtM])$group))

validation.score <- matrix(ncol=2,nrow=ncol(expr.test)) # create object to collect results

#Transpose for knn

training.data <- t(train.genes)

## set up matrix for ROC analysis

rocScore <- matrix(ncol=2,nrow=ncol(expr.test))

colnames(rocScore) <- c("label","predict")

labels <- testing.groups

for(i in 1:ncol(expr.test)){

if(labels[i] == 1)

rocScore[i,1] <- -1

else if(labels[i] == 2)

rocScore[i,1] <- 1

}

```

Predictions rely on one 'test' sample at a time and in this scenario the 'training data' to examine which 5 members of the data are closest to a given member of the test data. The predictions made in this process are recorded in the ```predict``` column of the ```rocScore``` matrix we set up above. In addition we record the specific array tested and whether the prediction was correct or not for each that array (these are recorded in columns1 & 2 of the ```validation.score``` matrix respectively). If the prediction is correct we increment the ```record``` variable by 1 and use this to calculate the percentage of correct classifications.

```{r eval=FALSE}

record <- 0

for (validation.array in 1:ncol(expr.test)){

test.data <-t(test.genes[,validation.array])

predict <- knn(train=training.data, test=test.data,cl=training.groups, k=knn.k)

if(predict==1){

rocScore[validation.array,2] <- -1

}

else if(predict==2){

rocScore[validation.array,2] <- 1

}

if (predict==testing.groups[validation.array]){

validation.score[validation.array,1] <- colnames(expr.test)[validation.array]

validation.score[validation.array,2] <- "Correct Prediction"

record <- record+1

}

else {

validation.score[validation.array,1] <- colnames(expr.test)[validation.array]

validation.score[validation.array,2] <- "Incorrect Prediction"

}

}

percentage <- 0

percentage <- ceiling((record/validation.array)*100)

```

We assess the results of the classification by ROC analysis in the code below. We classify the young as 'positive' and the old as 'negative' (these are arbitrary). To calculate the true positive rate (young classified as young) we first create a binary vector of true young. We then sum the ```predict``` values after filtering the ```rocScore``` dataframe by the binary vector.

Sensitivity is then calculated as the true positives divided by the actual number of young in the sample (i.e. the sum of the ```binary.values``` vector.

We calculate the false positives and false positive rate in a similar way to above, by inverting the binary labels. Finally we calculate the specificity.

```{r eval=FALSE}

threshold <- 0

# boolean vector of classes (young=TRUE; old=FALSE)

binary.labels <- rocScore[,1] == 1

# calculates total number of True Positives, i.e young classified as young

tp <- sum((rocScore[,2] > threshold) & binary.labels)

# calculates sensitivity i.e TP/total Positives from class labels

sensitivity <- tp/sum(binary.labels)

# calculates false positives for FPR/'1-specificity'

fp <- sum((rocScore[,2] > threshold ) & (!binary.labels))

# 1-specificity

fpRate <- fp/sum(!binary.labels)

sens <- round(sensitivity, digits = 3)

FPR <- round(fpRate, digits = 3)

spec <- 1-FPR

# Calculate area under the curve by using ROCR package

pred<-prediction(rocScore[,2], rocScore[,1])

auc<-attributes(performance(pred, 'auc'))$y.values[[1]]

```

Finally we write out a text file containing data on sensitivity and specificity.

```{r eval=FALSE}

write.table(validation.score,paste(Test_data,"_test_",Training_data,

"_Train__BatchAdj_150PS_",percentage,"SR_Sens=",sens,

"_Spec=",spec,".csv",sep=""),sep=',',quote=F)

```

**An explanation of simple rank-order (no linearity assumed) ranking to examine the performance of different signatures in clinical studies**

A number of researchers became very confused about linear versus non-linear classification model. We never utilised gene expression values and attempted to plot those values relative to age or any other clinical parameter in a linear manner. Instead, for case:control studies (matched for gender and chronological age) we created a cumulative **gene-ranking score** based on the individual rank order results from 150 genes in muscle as follows. If a gene was down-regulated in the original muscle age model e.g. between 25y and 65y, the sample with the highest expression was assigned a rank score of 1 and the subject with the lowest expression value was assigned the highest rank value. For genes up-regulated with age, the opposite ranking strategy was used. The median sum of these rank scores was calculated for each clinical sample and each gene provided equal weighting [1]. For the case-control analysis in our studies, gene selection was therefore also **entirely independent** of the subsequent clinical studies used i.e. it was hypothesis based, while the direction of regulation reflected regulation of that gene in healthy old muscle versus healthy young muscle. This gene ranking approach does not assume that there is a linear relationship between gene expression (or covariates and gene expression) and in fact only a small subset of the 150 genes correlate in a linear manner with chronological age. As such, the 150 genes can’t be used in a linear regression model as some have attempted e.g. Pilling and Melzer et al. The fact that we presented a non-linear model was clearly stated in the original article by Sood *et al* [1] and is implicit if the simple methods we used are understood. The ranking code is as follows:

---

title: "Gene ranking score calculation"

output: pdf_document

---

This code calculates the tissue ageing Gene Score for each sample, as an median of all of the selected genes (the classification gene-set). It is applied in one of two scenarios. 1) to samples where all individuals have the same chronological age (birth year) or 2) to contrast cases versus controls where the chronological age and gender is equal in both groups. The ranking score can be standardised to the total number of samples being ranked to compare across studies.

Thus, if a gene was downregulated with age in the discovery data set, then the sample with the lowest expression in this new data set will be marked youngest and if upregulated the sample with highest score will be the youngest

Setting up preliminaries like clearing workspace and setting study names

```{r eval=FALSE}

rm(list=ls())

name_of_study<-"Study"

signature<-"study_signature"

```

Loading data - normalised intensities matrix

```{r eval=FALSE}

expr.data <- read.table("Expression_matrix.txt",sep="\t",header=TRUE,row.names=1)

```

Loading the list of genes with annoteted directionality calculated in training dataset: downregulated with 'down' sign and upregulated genes with 'up' values

```{r eval=FALSE}

genesUD <- read.delim("list_of_genes.txt",sep='\t', header=T)

```

Select only the geneID and directionality column from the file

```{r eval=FALSE}

genesUD<- subset(genesUD,select=c("geneID","Directionality"))

```

Select genes that are present on a paltform

```{r eval=FALSE}

genesUD<-genesUD[genesUD$geneID %in% rownames(expr.data),]

```

Seperate up and down regulated genes

```{r eval=FALSE}

down <- subset(genesUD,genesUD$Directionality=="down")

dReg<-down$geneID

```

```{r eval=FALSE}

up <- subset(genesUD,genesUD$Directionality=="up")

uReg<-up$geneID

```

Calculates score of each gene for all samples

```{r eval=FALSE}

geneRank <- matrix(nrow=ncol(expr.data), ncol=length(genesUD[,1]) )

rownames(geneRank) <- colnames(expr.data)

colnames(geneRank) <- c(as.character(dReg), as.character(uReg))

```

DownRegulated genes with aging: Scores the sample with highest expression value as youngest and values it 1, then the next sample maximum/higher value as 2 and so on

```{r eval=FALSE}

for(i in 1: length(dReg)){

record <- 0

PS <- as.matrix(expr.data[which(rownames(expr.data)==dReg[i]),])

PS <- t(PS)

for(j in 1: ncol(expr.data)) {

maxIn <-which.max(PS)

maxIndex <- rownames(PS)[maxIn]

PS <- as.matrix(PS[-maxIn,])

sample <- which(maxIndex==rownames(geneRank))

geneRank[sample,which(colnames(geneRank)==dReg[i])] <- record

}

}

```

Upregulated genes with aging: Scores the sample with lowest expression value as youngest and values it 1, then the next sample minimum/lower value as 2 and so on.

```{r eval=FALSE}

for(i in 1: length(uReg)){

record <- 0

PS <- as.matrix(expr.data[which(rownames(expr.data)==uReg[i]),])

PS <- t(PS)

for(j in 1: ncol(expr.data)) {

minIn <-which.min(PS)

minIndex <- rownames(PS)[minIn]

PS <- as.matrix(PS[-minIn,])

sample <- which(minIndex==rownames(geneRank))

record <- record + 1

geneRank[sample,which(colnames(geneRank)==uReg[i])] <- record

}

}

```

Calculate median value for each sample based on their individual genes score

```{r eval=FALSE}

cumulative_geneRank<- apply(geneRank,1,median)

geneRank <- cbind(geneRank,cumulative_geneRank)

```

Write out the ranking matrix

```{r eval=FALSE}

write.table(geneRank, paste(name_of_study,"_genescore_ranking_basedon_",signature,

".csv",sep=""),sep=',',quote=F)

```

**The original code used to select the age-150 gene set is as follows:**

---

title: "Binary classifier reverse entry prototype"

output: pdf_document

---

We first clear the workspace and load required libraries.

```{r eval=FALSE}

rm(list=ls())

library(affy)

library(class)

library(limma)

```

The data is loaded into R and the .CEL suffix stripped from each of the cel file names.

```{r eval=FALSE}

dataIn <- ReadAffy(celfile.path='CEL_files_path')

# remove .CEL from file names

sampleNames(dataIn) <- sub('\\.CEL$', '', sampleNames(dataIn))

```

The phenotype data is loaded from an external file. This file contains the array identifiers as well as phenotype information such as group membership. We use the array name to ensure that the rows of the phenotype data match the order of the arrays.

```{r eval=FALSE}

phenoTable <- read.table('Phenodata/dataset.csv', sep=',', header=T)

rownames(phenoTable) <- phenoTable$array

#make a vector that matches the cel names of the arrays to the row names of the pheno data

mt<-match(sampleNames(dataIn), rownames(phenoTable))

# attach the pheno data

phenoData(dataIn) = new('AnnotatedDataFrame', data = phenoTable[mt,])

# create the exp.group vector in the correct order.

exp.group <- factor(as.numeric(phenoData(dataIn)$group))

```

We next set up parameters for the Leave One Out Cross Validation (LOOCV) procedure. These are the smallest and greatest number of genes to use in the K-nearest neighbour (KNN) classifier as well as the number of neighbours used.

```{r eval=FALSE}

# largest number of genes used to classify

max.gene <- 200

# lower bound of genes used to classify

min.gene <- 2

# number of KNN neighbours to consider

knn.k <- 5

```

The gene expression is normalised using the RMA algorithm and then centered and scaled prior to KNN.

```{r eval=FALSE}

eset.data <- rma(dataIn)

expr.data <- scale(exprs(eset.data), center=TRUE)

```

We'll be collecting data as a result of the classification. In this code below we set up the data structures we will use to collect this data.

```{r eval=FALSE}

#Initialise results vector

summary.vector <- vector(length=max.gene-min.gene+1)

names(summary.vector) <- min.gene:max.gene

# Initialise the scorematrix

scorematrix <- matrix(rep(0,nrow(expr.data)*2), nrow=nrow(expr.data), ncol=2)

rownames(scorematrix) <- rownames(expr.data)

# Score for each gene list (of n=200-2+1=199)

list.score <- vector(length=max.gene-min.gene + 1)

list.score[] <- 0

# Score for each PS used in list.opt(initilaised in the loop) classification

list.scorePS <- matrix(rep(0,nrow(expr.data)*2), nrow=nrow(expr.data), ncol=3)

colnames(list.scorePS) <- c("Appearance Count","Successful Predictions", "Success Ratio" )

rownames(list.scorePS) <- rownames(expr.data)

```

We now begin the selection of probesets for classification. This is a nested loop procedure. In the outer loop we hold out each array in turn. The middle loop uses KNN to examine the ability of selected probesets to classify the array. The innermost loop is used to select the probesets used in the middle loop.

The inner loop is used to select potentially useful probesets.Within the innermost loop an array is held out and limma is used to rank probesets in the remaining arrays by t-value. The top 200 probesets are taken forward in reverse order so that probesets with lower t-values are used for classification first. The ability of the selected probesets to classify the held out array is then tested using KNN. The results of the prediction and whether an individual probeset was used in that prediction are recorded. Each probeset is positively scored if it contributes to a correct classification.

This information is then used to select the top 150 performing probesets and these in turn go forward into a second KNN classification step on a second held out sample in the middle loop. Once again the classification performance of each probeset is recorded.

```{r eval=FALSE}

for (test.array in 1:ncol(expr.data)){

#Loop 2

for (list.opt in c(1:ncol(expr.data))[-test.array]){

#Loop 3. Testing predictive power of 2-200 genes

for (ngenes in min.gene:max.gene){

#If first time through loop for any holdout array get sig genes first

if (ngenes == min.gene){

# set design matrix

design <- cbind(1,exp.group[-c(test.array,list.opt)])

# fit models

fit <- lmFit(exprs(eset.data)[,-c(test.array, list.opt)], design = design)

# do empirical Bayes

fit2 <- eBayes(fit)

# get t stats, abs value

sig.genes <- abs(fit2$t[,2])

# sorted in order of abs t-value, decreasing

sig.genes <- sort(sig.genes, d=TRUE)

} # end eBayes here

# get top 200 sig probesets

top.200 <- sig.genes[c(1:max.gene)]

# REVERSE this list so lowest probesets go in first

top.200 <- rev(top.200)

# get the probesets to test this will vary with ngenes.

test.probes <- top.200[1:ngenes]

# get scaled expression data for ngenes (2 to 200), transpose for knn

candidate.genes <- expr.data[rownames(expr.data) %in% names(test.probes), ]

# transpose for knn (row-wise)

candidate.genes <- t(candidate.genes)

# take out the test.array and list.opt for training

training.data <- candidate.genes[-c(test.array, list.opt),]

# select test list.opt for testing

test.sample <- candidate.genes[list.opt, ]

# Prepare training, test and class data for knn

training.groups <- exp.group[-c(test.array, list.opt)]

# predict list.opt

predict <- knn(train=training.data, test=test.sample, cl=training.groups, k=knn.k)

# which probesets were used in this prediction

list.score.index <- which(rownames(list.scorePS) %in% names(test.probes))

# record the use (appearance) of that probeset

list.scorePS[list.score.index , 1] <- list.scorePS[list.score.index , 1] + 1

# print some details on classification

cat(paste("Predicting sample ", list.opt, "which is ", exp.group[list.opt],

" and leaving out sample", test.array,"\tusing",

ngenes, "genes \t"), sep="")

# so now we check result for list.opt for each ngenes

# if the prediction is right then add +1 to list.score position for ngenes

# this gives the list.size for a positive prediction

# if the prediction is right

# add +1 to the probe.performance position for included probes

if (predict==exp.group[list.opt]){

cat ("CORRECT\n")

list.score[ngenes-min.gene+1] <- list.score[ngenes-min.gene+1] + 1

list.scorePS[list.score.index,2] <- list.scorePS[list.score.index,2]+1

}

else {

cat ("Incorrect\n")

}

}

# END LOOP 3 - INNER LIST OPTIMISATION PREDICTIONS

# Select the first 150 genes from sig.genes for kNN prediction

test.length <- 150

# get the scaled expression data for the genes that correctly predicted

# test.array in loop 3

candidate.genes.testing <- expr.data[rownames(expr.data) %in%

names(sig.genes[1:test.length]),]

# transpose for knn

candidate.genes.testing <- t(candidate.genes.testing)

# take out the test.array for training data

training.data <- candidate.genes.testing[c(1:nrow(candidate.genes.testing))

[-c(test.array)],]

# get data for test.array only

test.sample <- candidate.genes.testing[test.array,]

# the true classes for training data only

training.groups <- as.factor(exp.group[-c(test.array)])

# do the prediction for the test.array

predict.testing <- knn(train=training.data, test=test.sample,

cl=training.groups, k=knn.k)

#print progress

cat ("Predicting TEST ARRAY with ", test.length," genes\t")

# if the prediction for test.array is right with ngenes

if (predict.testing==exp.group[test.array]){

cat ("CORRECT\n")

# and add +1 to those contributing genes in 1st column of the scorematrix

scorematrix[colnames(candidate.genes.testing),1] <-

scorematrix[colnames(candidate.genes.testing),1] + 1

}

else {

cat ("Incorrect\n")

}

# add +1 to the second column of score matrix for each candidate gene tested

# to count the number of times that gene is used in a prediction attempt

scorematrix[colnames(candidate.genes.testing),2] <-

scorematrix[colnames(candidate.genes.testing), 2] + 1

}

# END LOOP 2 (predicting test.array 1 with all poss list.opt)

}

# END LOOP 1

```

We record the probesets used in classification and the number of appearances each probeset makes in the classification step above. For each probeset the ratio of correct predictions to total appearances is calculated and finally the data is written out.

```{r eval=FALSE}

# keep only probesets used in predictions

# i.e. column 2 on scorematrix does not equal 0

scorematrix <- scorematrix[which(scorematrix[,2]!=0),]

colnames(scorematrix) <- c("Correct Preds", "Appearances")

# Sort according to scoring first column (correct classifications); best predictors first

scorematrix_App_Sorted <- scorematrix[order(scorematrix[,2], decreasing=TRUE),]

scorematrix_Appearance_data <- scorematrix_App_Sorted[,2]

# keep only probesets which appeared in top.200 lists

# i.e. column 1 on list.scorePS does not equal 0

list.scorePS <- list.scorePS[which(list.scorePS[,1]!=0),]

#calculates success ratio i.e correct predictions/ total appearance, for list.scorePS

list.scorePS[,3] <- list.scorePS[,2]/list.scorePS[,1]

#Sort list.scorePS according to first column (appearance count);

list.scorePS_Sorted <- list.scorePS[order(list.scorePS[,1], decreasing=TRUE),]

# write out data

write.table(scorematrix, "scorematrix_appearances_reverse_entry_prototype.txt",

sep="\t", quote=FALSE)

write.table(list.scorePS_Sorted,

"FinallistofclassifiyingPS_individualPS_appearances_successrate_

REVERSE_ENTRY_PROTOTYPE.txt",

sep="\t", quote=FALSE)

```

**Earlier methods presented online by Jacob and Speed.**

Jacob and Speed have posted multiple generations of critiques of our article – modifying the content and nature of their analysis as we pointed out fundamental flaws in their approach.

Early Analysis by Jacob and Speed re random sampling and muscle tissue ‘stacked the odds’ for their 2016 claimed performance of a ‘random’ 150 gene-set as a diagnostic for muscle age (old versus young samples). To following the computational cheat they implemented, you must examine 2 steps in their code and not just their main code. This check confirms what data is being ‘sampled’ to produce their gene lists. Notably, they did not record the gene-set identities and so no across cohort validation work was possible with any of their approaches.

The R session for the work by Jacob and Speed can be found here: <http://biorxiv.org/highwire/filestream/12735/field_highwire_adjunct_files/0/047050-1.gz>

**Their step 1)**

loadData.R: script where they load all the U133+2 gene-chip datasets from GEO/arrayexpress in their workspace and then create an rData object . It is this rData object that is used for their “random” sampling in the ageing-subsample.R script

If you look at this loadData.R script, on line 132 and line 133 they do this:

mads <- apply(X.GSE59880, 2, mad)

mad.ok <- names(mads)[mads > quantile(mads, 0.75)]

The mad function in R is defined as: "Compute the median absolute deviation, i.e., the (lo-/hi-) median of the absolute deviations from the median, and (by default) adjust by a factor for asymptotically normal consistency."

They save this mad.ok variable (**containing only the upper quantile of probe-sets that vary most with age**) in the object ageing-data.RData.

Step 2)

In the **ageing-subsample.R session**they load the **ageing-data.RData**from above and perform LOOCV.

At line 71 they do this:

**common.probesets** <- intersect(common.probesets,**mad.ok**)

and, then on line 133 they “randomly” sample from this the top 25% (common.probesets) list:

rand.sig <- sample(**common.probesets**, 150)

Anyone briefly inspecting their code will look at the ageing-subsample.R script thinking that the loadDat.R is just grabbing all the chip data. This approach to using R code and informatics is misleading in that is keeps the pre-filtering part of the code, within the loadData.R script and this was missed by reviewers, editors and bloggers. Thus, when using this code, they sample only from the most age-related genes in our original data – placing the cut-off below our prototype 670 genes, but including all of those genes and our 150 genes. The steps taken to obscure this step is rather disturbing.

**References**

1. Sood S, Gallagher IJ, Lunnon K, Rullman E, Keohane A, Crossland H, et al. A novel multi-tissue RNA diagnostic of healthy ageing relates to cognitive health status. Genome Biol [Internet]. Genome Biology; 2015;16:185. Available from: http://genomebiology.com/2015/16/1/185

2. Crossland H, Atherton PJJ, Strömberg A, Gustafsson T, Timmons JAA, Stro mberg A, et al. A reverse genetics cell-based evaluation of genes linked to healthy human tissue age. FASEB J [Internet]. 2016;31:1–14. Available from: http://europepmc.org/abstract/med/27698205

3. Sabatini DM. Twenty-five years of mTOR: Uncovering the link from nutrients to growth. Proc Natl Acad Sci [Internet]. 2017;0:201716173. Available from: http://www.pnas.org/lookup/doi/10.1073/pnas.1716173114

4. Yu L, Chibnik LB, Srivastava GP, Pochet N, Yang J, Xu J, et al. Association of Brain DNA Methylation in *SORL1* , *ABCA7* , *HLA-DRB5* , *SLC24A4* , and *BIN1* With Pathological Diagnosis of Alzheimer Disease. JAMA Neurol. 2015;72:15.

5. Kamboh MI, Demirci FY, Wang X, Minster RL, Carrasquillo MM, Pankratz VS, et al. Genome-wide association study of Alzheimer’s disease. Transl Psychiatry. 2012;2:e117.

6. Miller J a, Woltjer RL, Goodenbour JM, Horvath S, Geschwind DH. Genes and pathways underlying regional and cell type changes in Alzheimer’s disease. Genome Med. BioMed Central Ltd; 2013;5:48.

7. Bereczki E, Francis PT, Howlett D, Pereira JB, Höglund K, Bogstedt A, et al. Synaptic proteins predict cognitive decline in Alzheimer’s disease and Lewy body dementia. Alzheimer’s Dement [Internet]. 2016 [cited 2019 Apr 1];12:1149–58. Available from: https://linkinghub.elsevier.com/retrieve/pii/S1552526016302448

8. Huh S, Baek S-J, Lee KHK-H, Whitcomb DJ, Jo J, Choi S-M, et al. The reemergence of long-term potentiation in aged Alzheimer’s disease mouse model. Sci Rep. 2016;6:29152.

9. Xu J, de Winter F, Farrokhi C, Rockenstein E, Mante M, Adame A, et al. Neuregulin 1 improves cognitive deficits and neuropathology in an Alzheimer’s disease model. Sci Rep. 2016;6:31692.

10. Stricker R, Reiser G. Functions of the neuron-specific protein ADAP1 (centaurin-α1) in neuronal differentiation and neurodegenerative diseases, with an overview of structural and biochemical properties of ADAP1. Biol Chem. 2014;395:1321–40.

11. Kim JH, Song P, Lim H, Lee J-HJH, Lee J-HJH, Park SA, et al. Gene-Based Rare Allele Analysis Identified a Risk Gene of Alzheimer’s Disease. Arendt T, editor. PLoS One [Internet]. 2014 [cited 2019 Apr 1];9:e107983. Available from: http://www.ncbi.nlm.nih.gov/pubmed/25329708

12. Maurer B, Rumpf T, Scharfe M, Stolfa DA, Schmitt ML, He W, et al. Inhibitors of the NAD ^+^ -Dependent Protein Desuccinylase and Demalonylase Sirt5. ACS Med Chem Lett. 2012;3:1050–3.

13. Aird KM, Iwasaki O, Kossenkov A V., Tanizawa H, Fatkhutdinov N, Bitler BG, et al. HMGB2 orchestrates the chromatin landscape of senescence-associated secretory phenotype gene loci. J Cell Biol. 2016;215:325–34.

14. Yamanaka Y, Faghihi MA, Magistri M, Alvarez-Garcia O, Lotz M, Wahlestedt C. Antisense RNA controls LRP1 Sense transcript expression through interaction with a chromatin-associated protein, HMGB2. Cell Rep. 2015;11:967–76.

15. Li N, Hu P, Xu T, Chen H, Chen X, Hu J, et al. iTRAQ-based Proteomic Analysis of APPSw,Ind Mice Provides Insights into the Early Changes in Alzheimer’s Disease. Curr Alzheimer Res [Internet]. 2017 [cited 2019 Apr 1];14:1109–22. Available from: http://www.ncbi.nlm.nih.gov/pubmed/28730955

16. Sidhu VK, Huang BX, Desai A, Kevala K, Kim H-Y. Role of DHA in aging-related changes in mouse brain synaptic plasma membrane proteome. Neurobiol Aging [Internet]. 2016 [cited 2019 Apr 1];41:73–85. Available from: http://www.ncbi.nlm.nih.gov/pubmed/27103520

17. Crespo ÂC, Silva B, Marques L, Marcelino E, Maruta C, Costa S, et al. Genetic and biochemical markers in patients with Alzheimer’s disease support a concerted systemic iron homeostasis dysregulation. Neurobiol Aging [Internet]. 2014 [cited 2019 Apr 1];35:777–85. Available from: https://linkinghub.elsevier.com/retrieve/pii/S0197458013005460

18. Tan Q, Yalamanchili HK, Park J, De Maio A, Lu H-C, Wan Y-W, et al. Extensive cryptic splicing upon loss of RBM17 and TDP43 in neurodegeneration models. Hum Mol Genet [Internet]. 2016 [cited 2019 Apr 1];25:5083–93. Available from: https://academic.oup.com/hmg/article-lookup/doi/10.1093/hmg/ddw337

19. Lawrence I, Bene M, Nacarelli T, Azar A, Cohen JZ, Torres C, et al. Correlations between age, functional status, and the senescence-associated proteins HMGB2 and p16INK4a. GeroScience [Internet]. 2018 [cited 2019 Apr 1];40:193–9. Available from: http://www.ncbi.nlm.nih.gov/pubmed/29651745

20. Zirkel A, Nikolic M, Sofiadis K, Mallm J-P, Brackley CA, Gothe H, et al. HMGB2 Loss upon Senescence Entry Disrupts Genomic Organization and Induces CTCF Clustering across Cell Types. Mol Cell [Internet]. 2018 [cited 2019 Apr 1];70:730-744.e6. Available from: http://www.ncbi.nlm.nih.gov/pubmed/29706538

21. Li C, Yu L, Xue H, Yang Z, Yin Y, Zhang B, et al. Nuclear AMPK regulated CARM1 stabilization impacts autophagy in aged heart. Biochem Biophys Res Commun [Internet]. 2017 [cited 2019 Apr 1];486:398–405. Available from: http://www.ncbi.nlm.nih.gov/pubmed/28315332

22. Liu Y, Wang T, Ji YJ, Johnson K, Liu H, Johnson K, et al. A C9orf72–CARM1 axis regulates lipid metabolism under glucose starvation-induced nutrient stress. Genes Dev [Internet]. 2018 [cited 2019 Apr 1];32:1380–97. Available from: http://genesdev.cshlp.org/lookup/doi/10.1101/gad.315564.118

23. Park H, Kam T-I, Kim Y, Choi H, Gwon Y, Kim C, et al. Neuropathogenic role of adenylate kinase-1 in Aβ-mediated tau phosphorylation via AMPK and GSK3β. Hum Mol Genet [Internet]. 2012 [cited 2019 Apr 1];21:2725–37. Available from: http://www.ncbi.nlm.nih.gov/pubmed/22419736

24. Su Y, Wang P, Shen H, Sun Z, Xu C, Li G, et al. The protein kinase D1-mediated classical protein secretory pathway regulates the Ras oncogene-induced senescence response. J Cell Sci [Internet]. 2018 [cited 2019 Apr 1];131:jcs207217. Available from: http://www.ncbi.nlm.nih.gov/pubmed/29420297

25. Kumar V, Fleming T, Terjung S, Gorzelanny C, Gebhardt C, Agrawal R, et al. Homeostatic nuclear RAGE–ATM interaction is essential for efficient DNA repair. Nucleic Acids Res [Internet]. 2017 [cited 2019 Apr 1];45:10595–613. Available from: http://academic.oup.com/nar/article/45/18/10595/4079821

26. Cui L, Cai Y, Cheng W, Liu G, Zhao J, Cao H, et al. A Novel, Multi-Target Natural Drug Candidate, Matrine, Improves Cognitive Deficits in Alzheimer’s Disease Transgenic Mice by Inhibiting Aβ Aggregation and Blocking the RAGE/Aβ Axis. Mol Neurobiol [Internet]. 2017 [cited 2019 Apr 1];54:1939–52. Available from: http://www.ncbi.nlm.nih.gov/pubmed/26899576

27. Yang C, Li X, Mo Y, Liu S, Zhao L, Ma X, et al. β-Asarone Mitigates Amyloidosis and Downregulates RAGE in a Transgenic Mouse Model of Alzheimer’s Disease. Cell Mol Neurobiol [Internet]. 2016 [cited 2019 Apr 1];36:121–30. Available from: http://www.ncbi.nlm.nih.gov/pubmed/26271288

28. Zhang Y, Bharathi SS, Rardin MJ, Lu J, Maringer K V., Sims-Lucas S, et al. Lysine desuccinylase SIRT5 binds to cardiolipin and regulates the electron transport chain. J Biol Chem [Internet]. 2017 [cited 2019 Apr 1];292:10239–49. Available from: http://www.ncbi.nlm.nih.gov/pubmed/28458255

29. Sun X, Li PP, Zhu S, Cohen R, Marque LO, Ross CA, et al. Nuclear retention of full-length HTT RNA is mediated by splicing factors MBNL1 and U2AF65. Sci Rep [Internet]. 2015 [cited 2019 Apr 1];5:12521. Available from: http://www.nature.com/articles/srep12521

30. Gao X, Teng Y, Luo J, Huang L, Li M, Zhang Z, et al. The survival motor neuron gene *smn-1* interacts with the U2AF large subunit gene *uaf-1* to regulate *Caenorhabditis elegans* lifespan and motor functions. RNA Biol [Internet]. 2014 [cited 2019 Apr 1];11:1148–60. Available from: http://www.ncbi.nlm.nih.gov/pubmed/25483032

31. Szatmari EM, Oliveira AF, Sumner EJ, Yasuda R. Centaurin-α1-Ras-Elk-1 signaling at mitochondria mediates β-amyloid-induced synaptic dysfunction. J Neurosci [Internet]. 2013 [cited 2019 Apr 1];33:5367–74. Available from: http://www.jneurosci.org/cgi/doi/10.1523/JNEUROSCI.2641-12.2013

32. Zhao K, Shen C, Li L, Wu H, Xing G, Dong Z, et al. Sarcoglycan Alpha Mitigates Neuromuscular Junction Decline in Aged Mice by Stabilizing LRP4. J Neurosci [Internet]. 2018 [cited 2019 Apr 1];38:8860–73. Available from: http://www.ncbi.nlm.nih.gov/pubmed/30171091

33. Choi HY, Liu Y, Tennert C, Sugiura Y, Karakatsani A, Kröger S, et al. APP interacts with LRP4 and agrin to coordinate the development of the neuromuscular junction in mice. Elife [Internet]. 2013 [cited 2019 Apr 1];2:e00220. Available from: http://www.ncbi.nlm.nih.gov/pubmed/23986861

34. Spang N, Feldmann A, Huesmann H, Bekbulat F, Schmitt V, Hiebel C, et al. RAB3GAP1 and RAB3GAP2 modulate basal and rapamycin-induced autophagy. Autophagy [Internet]. 2014 [cited 2019 Mar 6];10:2297–309. Available from: http://www.ncbi.nlm.nih.gov/pubmed/25495476

35. Su M-Y, Morris KL, Kim DJ, Fu Y, Lawrence R, Stjepanovic G, et al. Hybrid Structure of the RagA/C-Ragulator mTORC1 Activation Complex. Mol Cell [Internet]. 2017 [cited 2019 Apr 1];68:835-846.e3. Available from: http://www.ncbi.nlm.nih.gov/pubmed/29107538

36. Bar-Peled L, Schweitzer LD, Zoncu R, Sabatini DM. Ragulator Is a GEF for the Rag GTPases that Signal Amino Acid Levels to mTORC1. Cell [Internet]. 2012 [cited 2018 Jan 24];150:1196–208. Available from: http://www.ncbi.nlm.nih.gov/pubmed/22980980

37. Xiao F-H, Chen X-Q, Yu Q, Ye Y, Liu Y-W, Yan D, et al. Transcriptome evidence reveals enhanced autophagy-lysosomal function in centenarians. Genome Res [Internet]. 2018 [cited 2019 Apr 1];28:1601–10. Available from: http://genome.cshlp.org/lookup/doi/10.1101/gr.220780.117

38. Shahmoradi A, Radyushkin K, Rossner MJ. Enhanced memory consolidation in mice lacking the circadian modulators Sharp1 and -2 caused by elevated Igf2 signaling in the cortex. Proc Natl Acad Sci U S A [Internet]. 2015 [cited 2019 Apr 1];112:E3582-9. Available from: http://www.pnas.org/lookup/doi/10.1073/pnas.1423989112

39. Verdaguer E, Brox S, Petrov D, Olloquequi J, Romero R, de Lemos ML, et al. Vulnerability of calbindin, calretinin and parvalbumin in a transgenic/knock-in APPswe/PS1dE9 mouse model of Alzheimer disease together with disruption of hippocampal neurogenesis. Exp Gerontol [Internet]. 2015 [cited 2019 Apr 1];69:176–88. Available from: http://www.ncbi.nlm.nih.gov/pubmed/26099796

40. Zallo F, Gardenal E, Verkhratsky A, Rodríguez JJ. Loss of calretinin and parvalbumin positive interneurones in the hippocampal CA1 of aged Alzheimer’s disease mice. Neurosci Lett [Internet]. 2018 [cited 2019 Apr 1];681:19–25. Available from: https://linkinghub.elsevier.com/retrieve/pii/S0304394018303628

41. Doens D, Valiente PA, Mfuh AM, X. T. Vo A, Tristan A, Carreño L, et al. Identification of Inhibitors of CD36-Amyloid Beta Binding as Potential Agents for Alzheimer’s Disease. ACS Chem Neurosci [Internet]. 2017 [cited 2019 Apr 1];8:1232–41. Available from: http://www.ncbi.nlm.nih.gov/pubmed/28150942

42. Vjetrovic J, Shankaranarayanan P, Mendoza-Parra MA, Gronemeyer H. Senescence-secreted factors activate Myc and sensitize pretransformed cells to TRAIL-induced apoptosis. Aging Cell [Internet]. 2014 [cited 2019 Apr 1];13:487–96. Available from: http://www.ncbi.nlm.nih.gov/pubmed/24589226

43. Hu C, Zhang Y, Tang K, Luo Y, Liu Y, Chen W. Downregulation of CITED2 contributes to TGFβ-mediated senescence of tendon-derived stem cells. Cell Tissue Res [Internet]. 2017 [cited 2019 Apr 1];368:93–104. Available from: http://www.ncbi.nlm.nih.gov/pubmed/28084522

44. Bigot N, Beauchef G, Hervieu M, Oddos T, Demoor M, Boumediene K, et al. NF-κB Accumulation Associated with COL1A1 Trans activators Defects during Chronological Aging Represses Type I Collagen Expression through a –112/–61-bp Region of the COL1A1 Promoter in Human Skin Fibroblasts. J Invest Dermatol [Internet]. 2012 [cited 2019 Apr 1];132:2360–7. Available from: http://www.ncbi.nlm.nih.gov/pubmed/22673730

45. Murray ER, Cameron AJM. Towards specific inhibition of mTORC2. Aging (Albany NY) [Internet]. 2017 [cited 2018 Jan 24];9:2461–2. Available from: http://www.ncbi.nlm.nih.gov/pubmed/29232655

46. Khoshnan A, Sabbaugh A, Calamini B, Marinero SA, Dunn DE, Yoo JH, et al. IKKβ and mutant huntingtin interactions regulate the expression of IL-34: implications for microglial-mediated neurodegeneration in HD. Hum Mol Genet [Internet]. 2017 [cited 2019 Apr 1];26:4267–77. Available from: http://www.ncbi.nlm.nih.gov/pubmed/28973132

47. Walker DG, Tang TM, Lue L-F. Studies on Colony Stimulating Factor Receptor-1 and Ligands Colony Stimulating Factor-1 and Interleukin-34 in Alzheimer’s Disease Brains and Human Microglia. Front Aging Neurosci [Internet]. 2017 [cited 2019 Apr 1];9:244. Available from: http://www.ncbi.nlm.nih.gov/pubmed/28848420

48. Novais A, Silva A, Ferreira AC, Falcão AM, Sousa N, Palha JA, et al. Adult Hippocampal Neurogenesis Modulation by the Membrane-Associated Progesterone Receptor Family Member Neudesin. Front Cell Neurosci [Internet]. 2018 [cited 2019 Apr 1];12:463. Available from: http://www.ncbi.nlm.nih.gov/pubmed/30534059

49. Severini C, Lattanzi R, Maftei D, Marconi V, Ciotti MT, Petrocchi Passeri P, et al. Bv8/prokineticin 2 is involved in Aβ-induced neurotoxicity. Sci Rep [Internet]. 2015 [cited 2019 Apr 1];5:15301. Available from: http://www.nature.com/articles/srep15301

50. Shin Y, Kim Y, Kim H, Shin N, Kim T, Kwon T, et al. RASAL3 preferentially stimulates GTP hydrolysis of the Rho family small GTPase Rac2. Biomed Reports [Internet]. 2018 [cited 2019 Apr 1]; Available from: http://www.spandidos-publications.com/10.3892/br.2018.1119

51. Bakkar N, Kovalik T, Lorenzini I, Spangler S, Lacoste A, Sponaugle K, et al. Artificial intelligence in neurodegenerative disease research: use of IBM Watson to identify additional RNA-binding proteins altered in amyotrophic lateral sclerosis. Acta Neuropathol [Internet]. 2018 [cited 2019 Apr 1];135:227–47. Available from: http://link.springer.com/10.1007/s00401-017-1785-8

52. Cheng Y, Liu P, Zheng Q, Gao G, Yuan J, Wang P, et al. Mitochondrial Trafficking and Processing of Telomerase RNA TERC. Cell Rep [Internet]. 2018 [cited 2019 Apr 1];24:2589–95. Available from: http://www.ncbi.nlm.nih.gov/pubmed/30184494

53. Sinkevicius KW, Morrison TR, Kulkarni P, Caffrey Cagliostro MK, Iriah S, Malmberg S, et al. *RNaseT2* knockout rats exhibit hippocampal neuropathology and deficits in memory. Dis Model Mech [Internet]. 2018 [cited 2019 Apr 1];11:dmm032631. Available from: http://www.ncbi.nlm.nih.gov/pubmed/29752287

54. Huang J, Liu P, Wang G. Regulation of mitochondrion-associated cytosolic ribosomes by mammalian mitochondrial ribonuclease T2 (RNASET2). J Biol Chem [Internet]. 2018 [cited 2019 Apr 1];293:19633–44. Available from: http://www.jbc.org/lookup/doi/10.1074/jbc.RA118.005433

55. XiYang Y-B, Wang Y-C, Zhao Y, Ru J, Lu B-T, Zhang Y-N, et al. Sodium Channel Voltage-Gated Beta 2 Plays a Vital Role in Brain Aging Associated with Synaptic Plasticity and Expression of COX5A and FGF-2. Mol Neurobiol [Internet]. 2016 [cited 2019 Apr 1];53:955–67. Available from: http://www.ncbi.nlm.nih.gov/pubmed/25575679

56. Stützer I, Selevsek N, Esterházy D, Schmidt A, Aebersold R, Stoffel M. Systematic Proteomic Analysis Identifies β-Site Amyloid Precursor Protein Cleaving Enzyme 2 and 1 (BACE2 and BACE1) Substrates in Pancreatic β-Cells. J Biol Chem [Internet]. 2013 [cited 2019 Apr 1];288:10536–47. Available from: http://www.ncbi.nlm.nih.gov/pubmed/23430253

57. Yaguchi H, Yabe I, Takahashi H, Watanabe M, Nomura T, Kano T, et al. Sez6l2 regulates phosphorylation of ADD and neuritogenesis. Biochem Biophys Res Commun [Internet]. 2017 [cited 2019 Apr 1];494:234–41. Available from: http://www.ncbi.nlm.nih.gov/pubmed/29032200

58. Boonen M, Staudt C, Gilis F, Oorschot V, Klumperman J, Jadot M. Cathepsin D and its newly identified transport receptor SEZ6L2 can modulate neurite outgrowth. J Cell Sci [Internet]. 2016 [cited 2019 Apr 1];129:557–68. Available from: http://www.ncbi.nlm.nih.gov/pubmed/26698217

59. Dumitriu A, Latourelle JC, Hadzi TC, Pankratz N, Garza D, Miller JP, et al. Gene Expression Profiles in Parkinson Disease Prefrontal Cortex Implicate FOXO1 and Genes under Its Transcriptional Regulation. Gibson G, editor. PLoS Genet [Internet]. 2012 [cited 2019 Apr 1];8:e1002794. Available from: http://www.ncbi.nlm.nih.gov/pubmed/22761592

60. Clarke T-K, Adams MJ, Davies G, Howard DM, Hall LS, Padmanabhan S, et al. Genome-wide association study of alcohol consumption and genetic overlap with other health-related traits in UK Biobank (N=112 117). Mol Psychiatry [Internet]. 2017 [cited 2019 Mar 6];22:1376–84. Available from: http://www.ncbi.nlm.nih.gov/pubmed/28937693

61. Cervetto C, Vergani L, Passalacqua M, Ragazzoni M, Venturini A, Cecconi F, et al. Astrocyte-Dependent Vulnerability to Excitotoxicity in Spermine Oxidase-Overexpressing Mouse. NeuroMolecular Med [Internet]. 2016 [cited 2019 Apr 1];18:50–68. Available from: http://link.springer.com/10.1007/s12017-015-8377-3

62. Costa E, Fernandes J, Ribeiro S, Sereno J, Garrido P, Rocha-Pereira P, et al. Aging is Associated with Impaired Renal Function, INF-gamma Induced Inflammation and with Alterations in Iron Regulatory Proteins Gene Expression. Aging Dis [Internet]. 2014 [cited 2019 Apr 1];5:356–65. Available from: http://www.ncbi.nlm.nih.gov/pubmed/25489488

63. Pasanen P, Myllykangas L, Pöyhönen M, Kiviharju A, Siitonen M, Hardy J, et al. Genetics of dementia in a Finnish cohort. Eur J Hum Genet [Internet]. 2018 [cited 2019 Mar 6];26:827–37. Available from: http://www.ncbi.nlm.nih.gov/pubmed/29476165

64. Brito-Moreira J, Lourenco M V., Oliveira MM, Ribeiro FC, Ledo JH, Diniz LP, et al. Interaction of amyloid-β (Aβ) oligomers with neurexin 2α and neuroligin 1 mediates synapse damage and memory loss in mice. J Biol Chem [Internet]. 2017 [cited 2019 Apr 1];292:7327–37. Available from: http://www.ncbi.nlm.nih.gov/pubmed/28283575

65. Sims R, Van Der Lee SJ, Naj AC, Bellenguez C, Badarinarayan N, Jakobsdottir J, et al. Rare coding variants in PLCG2, ABI3, and TREM2 implicate microglial-mediated innate immunity in Alzheimer’s disease. Nat. Genet. 2017.

66. Chang SL-Y, Chen S-Y, Huang H-H, Ko H-A, Liu P-T, Liu Y-C, et al. Ectopic Expression of Nolz-1 in Neural Progenitors Promotes Cell Cycle Exit/Premature Neuronal Differentiation Accompanying with Abnormal Apoptosis in the Developing Mouse Telencephalon. Chien C-T, editor. PLoS One [Internet]. 2013 [cited 2019 Apr 1];8:e74975. Available from: http://www.ncbi.nlm.nih.gov/pubmed/24073229

67. Glorioso C, Oh S, Douillard GG, Sibille E. Brain molecular aging, promotion of neurological disease and modulation by sirtuin 5 longevity gene polymorphism. Elsevier Inc.; 2011 [cited 2019 Apr 1];41. Available from: http://www.ncbi.nlm.nih.gov/pubmed/20887790

68. Reiser G, Bernstein H-G. Altered expression of protein p42IP4/centaurin-alpha 1 in Alzheimer’s disease brains and possible interaction of p42IP4 with nucleolin. Neuroreport. 2004;15:147–8.

69. Taru H, Iijima K-I, Hase M, Kirino Y, Yagi Y, Suzuki T. Interaction of Alzheimer’s beta -amyloid precursor family proteins with scaffold proteins of the JNK signaling cascade. J Biol Chem [Internet]. 2002 [cited 2019 Apr 1];277:20070–8. Available from: http://www.jbc.org/lookup/doi/10.1074/jbc.M108372200

70. Taniguchi N, Carames B, Ronfani L, Ulmer U, Komiya S, Bianchi ME, et al. Aging-related loss of the chromatin protein HMGB2 in articular cartilage is linked to reduced cellularity and osteoarthritis. Proc Natl Acad Sci [Internet]. 2009 [cited 2019 Apr 1];106:1181–6. Available from: http://www.pnas.org/cgi/doi/10.1073/pnas.0806062106

71. Seshadri S, Fitzpatrick AL, Ikram MA, DeStefano AL, Gudnason V, Boada M, et al. Genome-wide Analysis of Genetic Loci Associated With Alzheimer Disease. JAMA [Internet]. 2010 [cited 2019 Apr 1];303:1832. Available from: http://www.ncbi.nlm.nih.gov/pubmed/20460622

72. Fonseca M, Soriano E. Calretinin-immunoreactive neurons in the normal human temporal cortex and in Alzheimer’s disease. Brain Res [Internet]. 1995 [cited 2019 Apr 1];691:83–91. Available from: http://www.ncbi.nlm.nih.gov/pubmed/8590068

73. Coraci IS, Husemann J, Berman JW, Hulette C, Dufour JH, Campanella GK, et al. CD36, a class B scavenger receptor, is expressed on microglia in Alzheimer’s disease brains and can mediate production of reactive oxygen species in response to beta-amyloid fibrils. Am J Pathol [Internet]. 2002 [cited 2019 Apr 1];160:101–12. Available from: http://www.ncbi.nlm.nih.gov/pubmed/11786404

74. Micheau O. Cellular FLICE-inhibitory protein: an attractive therapeutic target? Expert Opin Ther Targets [Internet]. 2003 [cited 2019 Apr 1];7:559–73. Available from: http://www.ncbi.nlm.nih.gov/pubmed/12885274

75. Giampietri C, Petrungaro S, Coluccia P, Antonangeli F, Giannakakis K, Faraggiana T, et al. c-Flip overexpression affects satellite cell proliferation and promotes skeletal muscle aging. Cell Death Dis [Internet]. 2010 [cited 2019 Apr 1];1:e38–e38. Available from: http://www.ncbi.nlm.nih.gov/pubmed/21364645

76. Kung AWC, Xiao S-M, Cherny S, Li GHY, Gao Y, Tso G, et al. Association of JAG1 with Bone Mineral Density and Osteoporotic Fractures: A Genome-wide Association Study and Follow-up Replication Studies. Am J Hum Genet [Internet]. 2010 [cited 2019 Apr 1];86:229–39. Available from: http://www.ncbi.nlm.nih.gov/pubmed/20096396

77. Huang L, Wu X, Jiang H, Gao P, Kuang C, Wang K, et al. Aging reduces susceptibility of vascular smooth muscle cells to H2O2-induced apoptosis through the down-regulation of Jagged1 expression in endothelial cells. Int J Mol Med [Internet]. 2011 [cited 2019 Apr 1];28:207–13. Available from: http://www.ncbi.nlm.nih.gov/pubmed/21491077

78. Wu X, Zhou Q, Huang L, Sun A, Wang K, Zou Y, et al. Ageing-exaggerated proliferation of vascular smooth muscle cells is related to attenuation of Jagged1 expression in endothelial cells. Cardiovasc Res [Internet]. 2008 [cited 2019 Apr 1];77:800–8. Available from: http://www.ncbi.nlm.nih.gov/pubmed/18079106

79. Varela I, Cadiñanos J, Pendás AM, Gutiérrez-Fernández A, Folgueras AR, Sánchez LM, et al. Accelerated ageing in mice deficient in Zmpste24 protease is linked to p53 signalling activation. Nature [Internet]. 2005 [cited 2019 Apr 1];437:564–8. Available from: http://www.ncbi.nlm.nih.gov/pubmed/16079796

80. Jacinto E, Facchinetti V, Liu D, Soto N, Wei S, Jung SY, et al. SIN1/MIP1 Maintains rictor-mTOR Complex Integrity and Regulates Akt Phosphorylation and Substrate Specificity. Cell [Internet]. 2006 [cited 2018 Jan 24];127:125–37. Available from: http://www.ncbi.nlm.nih.gov/pubmed/16962653

81. Patel A, Rees SD, Kelly MA, Bain SC, Barnett AH, Thalitaya D, et al. Association of variants within APOE, SORL1, RUNX1, BACE1 and ALDH18A1 with dementia in Alzheimer’s disease in subjects with Down syndrome. Neurosci Lett [Internet]. 2011 [cited 2019 Apr 1];487:144–8. Available from: http://www.ncbi.nlm.nih.gov/pubmed/20946940

82. Romano GH, Harari Y, Yehuda T, Podhorzer A, Rubinstein L, Shamir R, et al. Environmental Stresses Disrupt Telomere Length Homeostasis. Zhou J-Q, editor. PLoS Genet [Internet]. 2013 [cited 2019 Apr 1];9:e1003721. Available from: http://www.ncbi.nlm.nih.gov/pubmed/24039592

83. Matsuo A, Walker DG, Terai K, McGeer PL. Expression of CD43 in human microglia and its downregulation in Alzheimer’s disease. J Neuroimmunol [Internet]. 1996 [cited 2019 Apr 1];71:81–6. Available from: http://www.ncbi.nlm.nih.gov/pubmed/8982106

84. Speed T. Statistical analysis of gene expression microarray data. CRC Press; 2003.
